# Is it Necessary to Send Clinically Diagnosed Benign Skin and Subcutaneous Lesions Excised Under Local Anesthesia for Routine Histopathological Examination?

**DOI:** 10.7759/cureus.17194

**Published:** 2021-08-15

**Authors:** Cyril Joseph Milton, Karthika Ganesamoorthy, Dharanya GS, Amudhan Kannan, Chellappa Vijayakumar, Bheemanathi Hanuman Srinivas, Sudharsanan Sundaramurthi

**Affiliations:** 1 Medicine, Jawaharlal Institute of Postgraduate Medical Education and Research, Puducherry, IND; 2 Preventive and Social Medicine, Jawaharlal Institute of Postgraduate Medical Education and Research, Puducherry, IND; 3 Otorhinolaryngology, Sri Venkateshwaraa Medical College Hospital and Research Centre, Puducherry, IND; 4 Surgery, Jawaharlal Institute of Postgraduate Medical Education and Research, Puducherry, IND; 5 Pathology, Jawaharlal Institute of Postgraduate Medical Education and Research, Puducherry, IND

**Keywords:** benign lesions, dermoid cyst, histopathological examination, ganglion, lipoma, sebaceous cyst, minor skin surgery

## Abstract

Background

The common benign surgical lesions of skin and subcutaneous tissues like lipoma and sebaceous cysts are diagnosed clinically and treated by surgical excision under local anesthesia. The occurrence of malignancy in these lesions is extremely low, and routine histopathological examination (HPE) adds to increased costs and workload on pathologists. This study was undertaken to estimate the clinical concordance and the frequency of malignancy in these lesions.

Methods

A total of 1,815 HPE reports of clinically benign skin and subcutaneous lesions excised under local anesthesia from January 2014 to December 2018 were studied.

Results

Lipoma (31.3%) and sebaceous cyst (29.9%) were the common clinical diagnosis in our study. The clinical accuracy in the diagnosis of lipoma was 88.6%, and for sebaceous cyst, it was 72.7%. There were six reports of malignancy in our study from the clinically diagnosed benign skin and subcutaneous lesions (0.33%). None of the cases of lipoma and the sebaceous cyst had a malignancy in the final histopathology report. The frequency of malignancy in the rest of the lesions is 0.85% (six out of 699 cases); (p-value: 0.003).

Discussion

In the absence of red flag signs, lesions like lipoma, sebaceous cyst, corn, and callus can be discarded without an HPE. We advocate routine HPE of other solid lesions, cystic lesions with solid areas, and pigmented or ulcerated lesions, as the clinical concordance is low and there is a significant occurrence of malignancy in these lesions.

## Introduction

Lesions like lipoma, sebaceous cysts, and dermoid cysts are commonly occurring benign lesions of skin and subcutaneous tissues. These lesions are often diagnosed based on clinical examination findings. The treatment of these benign conditions is surgical excision done under local anesthesia. After complete excision, the specimens are routinely sent for histopathological examination (HPE) to confirm whether it is a benign or malignant lesion. However, the rate of malignancy in these lesions is extremely low. The incidence of malignancy in some conditions like callus and corns is virtually nil.

Moreover, in our experience, a few patients do not follow up after surgical excision of these lesions, and the HPE reports are not studied by the surgeons subsequently. The “send all the excised” practice is historic surgical teaching where surgeons were encouraged to routinely send all excised specimens for histological examination, irrespective of clinical certainty for definitive documentation, and thus avoid potential medico-legal action [1]. There are published studies in the literature that reported rare cases of melanoma, squamous cell carcinoma, and basal cell carcinoma reported in sebaceous cysts [2-6].

Though some surgeons send every specimen for histopathological review, others advise sending specimens for HPE only if an unusual finding was encountered [7]. The role of routine HPE of specimens had been investigated in other subspecialties such as colorectal surgery for intestinal polyps, ENT for tonsillectomy specimens, and hepatobiliary surgery for cholecystectomy specimens, where HPE is suggested only in those with risk factors or uncertainty or with uncommon findings [8,9]. So, a disjunction exists in the opinion of whether all such specimens should be sent for routine pathologic evaluation. However, no such studies have been performed involving various benign swelling of the skin and subcutaneous tissue.

Therefore, this retrospective study was done to study the concordance between clinical diagnosis and histopathology report to address this controversial topic and estimate the malignancy frequency reported in clinically diagnosed benign skin and subcutaneous lesions.

## Materials and methods

This is a retrospective descriptive study conducted in the Department of Surgery, Jawaharlal Institute of Postgraduate Medical Education and Research (JIPMER), Puducherry. Institute ethics clearance was obtained (JIP/IEC/2019/0107). All patients diagnosed with benign skin and subcutaneous lesions treated by excision under local anesthesia in the minor operation theatre from January 2014 to December 2018 were included. The lesions were diagnosed as benign by their indolent nature, without fixation to surrounding structures, and with a smaller size that can be safely excised under local anesthesia as a daycare procedure. Patients who presented with skin lesions that were excised as a part of a diagnostic evaluation, like lymph node swellings and breast lumps, were excluded.

We retrieved the details, such as the clinical diagnosis of benign skin and subcutaneous lesions, from the minor operation theatre register. The hospital information system (HIS) portal of our institute was used to access the HPE reports of all these cases. All the pertinent information was entered in Microsoft Excel. The hospital case sheets of those cases that were reported as malignant were collected from our institute's medical records department for a detailed study of the clinical picture.

Statistical analysis was performed using STATA/MP 14.0. The distribution of continuous data was expressed in mean and standard deviation. The distribution of categorical data was expressed in frequencies and percentages. The comparison of the percentage of malignancy between categorical variables was carried out using Fischer exact test. All statistical analyses were carried out at a 5% significance level, and p-value <0.05 was considered significant.

## Results

A total of 3,800 clinically diagnosed benign skin and subcutaneous lesions were excised under local anesthesia in the Surgery Minor OT in the study period, January 2014-December 2018. Out of these, 1815 lesions were sent for histopathological examination; therefore, only 1815 were included in the study. The mean age of the study group is 40±14 years. In our study, 1020 (56.2%) were male patients. Table 1 shows the various clinical diagnoses in our study.

**Table 1:** The various clinical diagnoses of skin and subcutaneous lesions excised under local anesthesia.

S. No	Clinical diagnosis	Number (%)
1.	Lipoma	568 (31.3)
2.	Sebaceous cyst	542 (29.9)
3.	Dermoid cyst	269 (14.8)
4.	Ganglion	98 (5.4)
5.	Papilloma	78 (4.3)
6.	Corn	51 (2.8)
7.	Calcinosis cutis	39 (2.2)
8.	Neurofibroma	38 (2.1)
9.	Pyogenic granuloma	36 (2.0)
10.	Others	96 (5.3)
	Total	1815

The most common lesion sent for histopathology in the study period was lipoma in 568 (31.3%) cases, followed by the sebaceous cyst and dermoid cyst in 542 (29.86%) and 269 (14.83%) cases, respectively. Other common clinically diagnosed lesions are ganglion in 98 (5.4%) cases, papilloma in78 (4.3%) cases, corn in51 (2.8%) cases, and neurofibroma in 38 (2%) cases. Less commonly lesions sent for histopathological examination include fibroma, corn, callus, granuloma, angiokeratoma, keloid, and calcinosis cutis. A total of six malignancies were reported in our study, with an incidence of 0.33% in clinically diagnosed benign skin and subcutaneous lesions. There are no reports of malignancies reported in cases with the clinical diagnosis of lipoma and sebaceous cyst. Excluding lipoma and sebaceous cyst, the frequency of malignancy in the rest of the lesions is 0.85% (six out of 699 cases; p = 0.003). The clinically diagnosed benign lesions that turned out to be malignant on HPE are listed in Table 2.

**Table 2:** Clinical diagnosis of lesions that were malignant on histopathological examination.

S. No.	Malignancy in histopathological examination	Clinical diagnosis
1	Mucoepidermoid carcinoma	Dermoid cyst
2	Malignant trichilemmal cyst	Dermoid cyst
3	Malignant melanoma	Ganglion
4	Synovial carcinoma	Neurofibroma
5	Verrucous carcinoma	Pyogenic granuloma
6	Synovial sarcoma	Dermoid cyst

The frequency of malignancy in the final histopathology report compared to the clinical diagnosis is presented in Table 3.

**Table 3:** Frequency of malignancy in the final histopathology report compared to the clinical diagnosis. *Fischer exact test.

Clinical diagnosis	Benign	Malignant	p-value*
Lipoma and sebaceous cyst	1110	0	0.003
Others	699	6	

**Figure 1:**
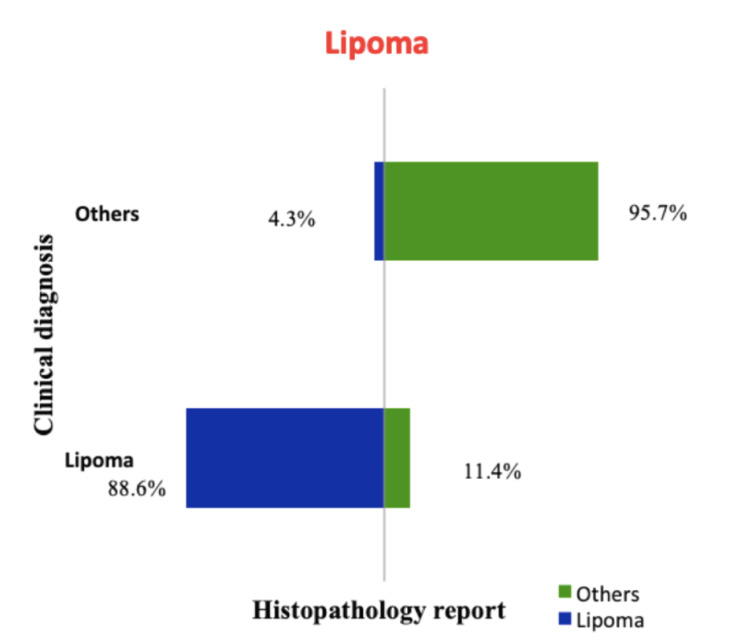
Concordance between clinical diagnosis and histopathology report for lipoma.

**Figure 2:**
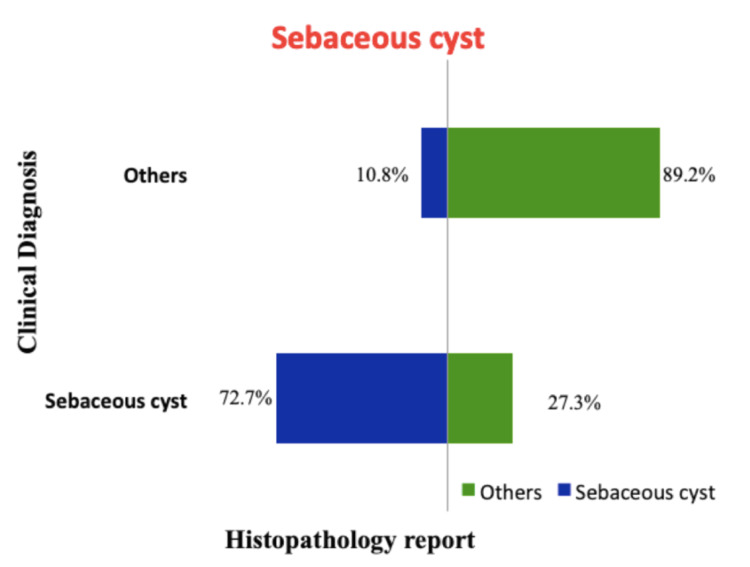
Concordance between clinical diagnosis and histopathology report for sebaceous cyst.

**Figure 3:**
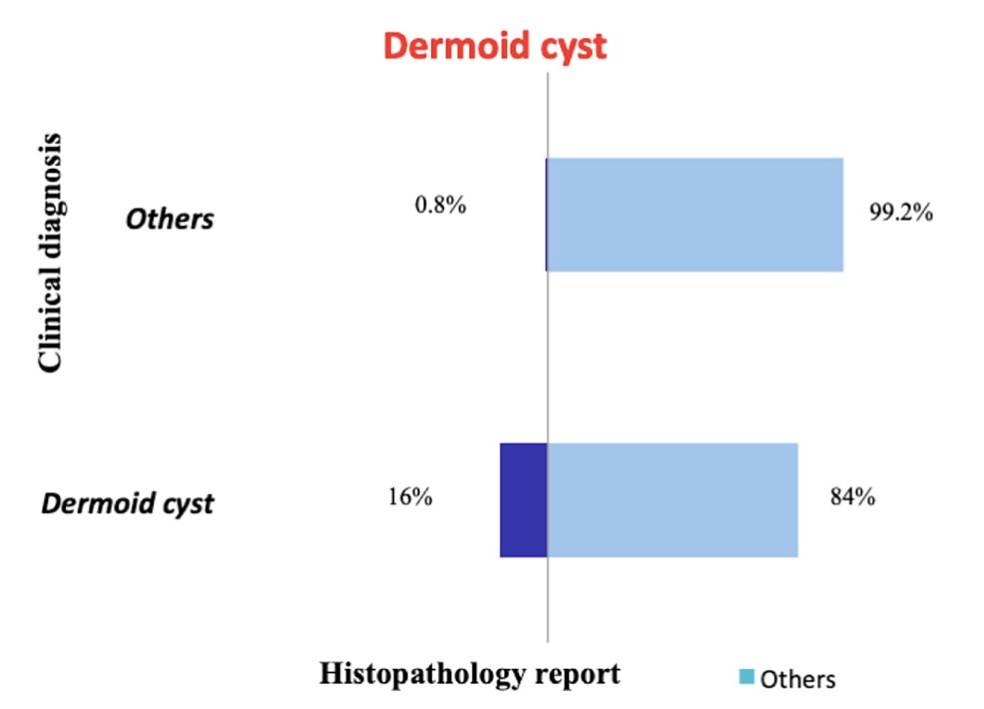
Concordance between clinical diagnosis and histopathology report for dermoid cyst.

**Figure 4:**
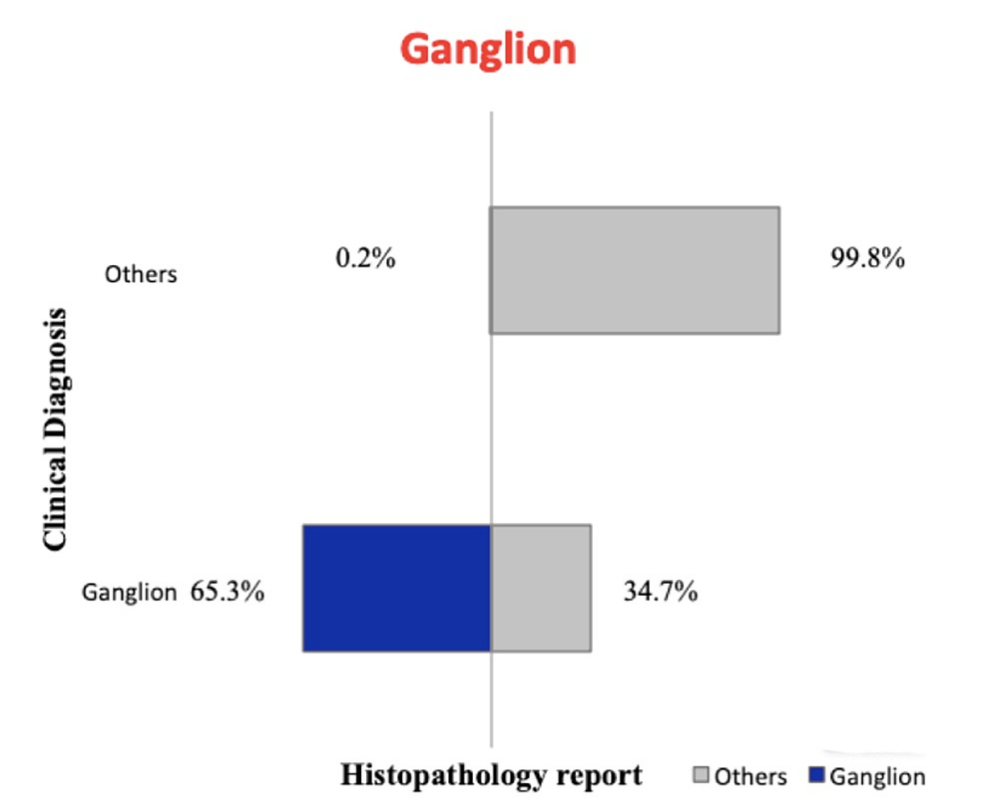
Concordance between clinical diagnosis and histopathology report for ganglion.

The concordance between the clinical diagnosis and histopathology report among the common lesions is the highest for lipoma with a concordance of 88.6% and the least for dermoid cyst at 16% clinical concordance. The clinical concordances of the lesions are shown in Figures 1, 2, 3, 4. A total of 41 phaeohyphomycotic cysts were reported in histopathology from various clinical diagnoses in our study.

## Discussion

Various studies have shown that about 25%-40% of all laboratory tests are unnecessary and are done mainly for completion of diagnosis, meeting the patients' expectations, and fear of medico-legal consequences [1]. The cost of histopathological studies and the work burden on pathologists are rising day by day. This also leads to a delay in the reporting of more important malignant cases. This study was done to estimate the clinical concordance and incidence of malignancies reported in clinically diagnosed benign skin and subcutaneous lesions.

This was a retrospective study of 1,815 histopathological reports of clinically diagnosed benign skin and subcutaneous lesions that were excised under local anesthesia. The common lesions examined in this study include lipoma, sebaceous cysts, dermoid cysts, and ganglion. Lipoma and sebaceous cysts are the common benign skin and subcutaneous lesions excised under local anesthesia in various studies [10].

The clinical diagnosis was concordant with the final histopathological report in 88.6% of cases of lipoma. For sebaceous cyst, the concordance was 72.7%, which was less compared to the clinical accuracy of 81.9% by the assessing surgeons in the study by Apollos et al. [11]. In this study, the clinical diagnostic accuracy for a ganglion is fair with 65.3% concordance, and for dermoid cyst, it is extremely low at 16%. This suggests that the dermoid cysts were the most commonly used clinical diagnosis for other non-dermoid lesions. This could be because the dermoid cysts have non-specific clinical findings that can be seen in other benign lesions and because the surgical trainees were overemphasized in their curriculum about dermoid cysts than the other clinically mimicking lesions. There were six histopathological reports of malignancy in our study from the clinically diagnosed benign skin and subcutaneous lesions (0.33%). In the study by Gargya et al. on histopathology of sebaceous cysts, the occurrence of malignancy was 0.3% [12]. In our study, none of the clinically diagnosed lipoma and sebaceous cysts had malignancy in the final histopathological report.

Three malignancies were reported from clinically diagnosed dermoid cysts (1.12%) and one malignancy each from ganglion (1.02%), neurofibroma (2.63%), and pyogenic granuloma (2.78%). The malignancies identified in our study are mucoepidermoid carcinoma, soft tissue sarcoma, and malignant trichilemmal cyst, all three diagnosed clinically as a dermoid cyst; a malignant melanoma diagnosed as ganglion of the ankle, a verrucous carcinoma diagnosed clinically as pyogenic granuloma, and a synovial sarcoma with a clinical diagnosis of benign neurofibroma.

For other clinically diagnosed benign lesions like papilloma, corn, callus, calcinosis cutis, and hemangioma, the histopathology did not show any malignant condition that requires further management. Histopathological diagnosis of the phaeohyphomycotic cyst was reported in 41 (2.25%) cases in our study. Dermoid cyst and ganglion were the commonly used clinical diagnosis for these fungal cysts. All the phaeohyphomycotic cysts were excised in total and did not require further treatment.

The incidence of malignancy from the clinically diagnosed benign skin and subcutaneous lesions becomes significant only when lipoma and sebaceous cysts have been excluded clinically, six malignancies out of 699 lesions (p-value: 0.003). Morritt et al. described red flag features like rapid growth and ulceration that raise the suspicion of malignancy in a sebaceous cyst [13]. Further, the sebaceous cysts can be incised routinely after their removal and looked for cheesy pultaceous material; the presence of solid areas within the cyst should strongly raise the suspicion of malignancy.

## Conclusions

Routine HPE of lesions like lipoma, sebaceous cyst, corn, and callus can be deferred if no pathognomic signs of malignancy are present both pre-operatively and intra-operatively. Based on this study, we advocate the routine use of histological examination in all other solid lesions, cystic lesions with solid areas within it, pigmented lesions, ulcerated lesions, and other lesions where there is a strong suspicion of malignancy intra-operatively. Healthcare resources are limited, especially in developing countries, and therefore must be used wisely. This approach can lessen the workload on pathologists, may indirectly facilitate faster reporting of important malignant samples, and considerable expenditure on processing these samples can be curtailed.
